# The Vascular SIRTainty

**DOI:** 10.18632/aging.100161

**Published:** 2010-06-26

**Authors:** Zhihong Yang, Xiu-Fen Ming

**Affiliations:** Vascular Biology, Department of Medicine, Division of Physiology, Faculty of Science, University of Fribourg, 1700 Fribourg, Switzerland

**Keywords:** 06/22/10; accepted: 06/24/10; published on line: 06/26/10

Atherosclerotic cardiovascular disease remains globally the main cause of death and morbidity [1]. The underlying mechanism of atherogenesis is multifactorial, involving functional changes of vascular cells including endothelial cells, smooth muscle cells, adventitial, and perivascular cells, and circulating cells including platelets and inflammatory cells [2]. Clinical studies demonstrate that endothelial dysfunction reflected by decreased bioavailability of endothelial nitric oxide (NO) derived from endothelial NO-synthase (eNOS) is not only associated with atherosclerosis and risk factors such as hypercholesterolemia, diabetes, advanced age, etc, but also predicts the future clinical outcomes of patients [3]. The firm evidence for the role of eNOS in anti-atherogenesis stems from early experimental studies demonstrating accelerated atherosclerosis in *ApoE^-/-^/eNOS^-/-^* double knockout mice compared to *ApoE^-/-^* mice [4]. Endothelial dysfunction is therefore widely accepted as a fundamental pathophysiological mechanism linking various cardiovascular risk factors to atherosclerosis. The dysfunctional endothelial cells express elevated adhesion molecules such as VCAM-1 and ICAM-1, thereby promoting monocyte-endothelial interaction subsequently transmigration into the intima, where the monocytes mature to macrophages, take up lipids, become foam cells, leading to atheroma plaque formation [5].

It is of note that at early to middle stages of atherosclerosis and advanced age, eNOS protein level in the vasculature is not decreased and even upregulated [6,7]. Much effort in the past has therefore been made to investigate mechanisms that regulate NO bioavailability at the eNOS enzymatic level. Among other mechanisms, an imbalance between eNOS activity and oxidative stress seems critical for endothelial dysfunction under the conditions [8]. Alone this line, strategies designed to inhibit oxidative stress or to enhance eNOS enzyme activity are considered promising therapeutic possibilities for prevention and/or treatment of atherosclerosis. One of the targets which fulfils the therapeutic purpose is the NAD^+^-dependent histone deacetylase sirtuin-1 (silent mating type information regulation 2 homolog, SIRT1), whose activation has been shown to exert numerous beneficial effects in many aspects including life-span expansion, inhibition of endothelial senescence, inflammation, oxidative stress, and beneficial regulation of carbohydrate and lipid metabolism [9].

Most recent studies demonstrate that SIRT1 interacts directly with eNOS, causes deacetylation of the enzyme at lysines 496 and 506 and posttranslationally enhances eNOS activity [10, 11]. The relationship between SIRT1-eNOS signaling and atherosclerosis has been implicated recently by Chen and colleagues [11], who show that SIRT1 level is higher in descending thoracic aortas (atherosclerotic resistant region) than aortic arches (atherosclerotic prone region) of C57BL6 mice. Importantly, another study by Zhang and colleagues demonstrate that endothelial specific over-expression of SIRT1 decreases atherosclerosis in *ApoE^-/-^*mice [12]. The present study by Stein and colleagues [13] take further the "loss-of-function" experimental approach by using *SIRT1^+/-^/ApoE^-/-^* mice to determine the role of endogenous SIRT1 on atherogenesis and endothelial function. They show in a recently published study reduced atherosclerotic lesion formation and inhibition of foam cell formation in *SIRT1^+/-^/ApoE^-/-^* mice as compared to control *ApoE^-/-^* mice [14], confirming the atheroprotective role of SIRT1. In the current study, the authors provide further information showing more pronounced plaque ICAM-1 and VCAM-1 levels in *SIRT1^+/-^/ApoE^-/-^* mice and endothelial expression of ICAM-1 and VCAM-1 upon *in vivo* challenge with lipopolysaccharide to induce systemic inflammation. The inhibitory role of SIRT1 in endothelial inflammatory responses is further confirmed in cultured human aortic endothelial cells in response to TNF-α. An increase in reactive oxygen species (ROS) in cells in which *SIRT1* is silenced by siRNA has been also demonstrated. The results support the findings by various studies demonstrating anti-inflammatory and anti-oxidative effects of SIRT1 in endothelial cells. Surprisingly, no difference in endothelium-dependent relaxations and eNOS-S1177 phosphorylation, an activating mechanism of the enzyme between *SIRT1^+/-^/ApoE^-/-^* and *ApoE^-/-^* mice is observed. It seems that there is discrepancy between this study and the study by Zhang, et al. [12] who report improved endothelium-dependent relaxations in endothelial specific *SIRT1* transgenic mice. The discrepancy may be explained by different experimental conditions between the two studies. In the study by Zhang, endothelial function is investigated in wild type and endothelial specific *SIRT1 *transgenic mice fed high-fat-diet (HFD) for a relatively longer period i.e. 6 months, while the atherosclerotic burden, unfortunately not the endothelial function, is studied in *ApoE^-/-^* and *ApoE^-/-^* endothelial *SIRT1* transgenic mice on HFD for 10 weeks, a protocol which is similar to that used in the study by Stein. It seems that the food utilized in the two studies is also different. Moreover, the protection of endothelial function observed in the study by Zhang could be due to an over-expression of the transgene *SIRT1* which may exert much stronger effect than single *SIRT1* allele deletion model used in the study by Stein. Another possible explanation could be that the whole body single allele deletion of *SIRT1* may readily affect other cell functions such as monocytes/macrophages as demonstrated by the authors in the same series of study [14], but may not be sufficient to affect eNOS activity. This concern could be addressed by determining whether eNOS acetylation level is indeed altered by single allele deletion of *SIRT1* in their mouse model in the future experiments. Nevertheless, the study by Stein and colleagues **certainly** further supports the hypothesis that SIRT1 may be a promising therapeutic target to prevent or treat atherosclerosis.

## References

[1] Lopez AD, Mathers CD, Ezzati M, Jamison DT, Murray CJ (2006). Lancet.

[2] Yang Z, Ming XF (2006). Clin Med Res.

[3] Schachinger V, Britten MB, Zeiher AM (2000). Circulation.

[4] Knowles JW, Reddick RL, Jennette JC (2000). J Clin Invest.

[5] Libby P, Ridker PM, Hansson GK (2009). J Am Coll Cardiol.

[6] Ming XF, Barandier C, Viswambharan H (2004). Circulation.

[7] van der Loo B, Labugger R, Skepper JN (2000). J Exp Med.

[8] Stocker R, Keaney JF Jr (2004). Physiol Rev.

[9] Finkel T, Deng CX, Mostoslavsky R (2009). Nature.

[10] Mattagajasingh I, Kim CS, Naqvi A (2007). Proc Natl Acad Sci U S A.

[11] Chen Z, Peng IC, Cui X, Li YS, Chien S, Shyy JY (2010). Proc Natl Acad Sci U S A.

[12] Zhang QJ, Wang Z, Chen HZ (2008). Cardiovasc Res.

[13] Stein S, Schäfer N, Breitenstein A (2010). Aging.

[14] Stein S, Lohmann C, Schafer N (2010). Eur Heart J.

